# Search for genetic factors predisposing to atherogenic dyslipidemia

**DOI:** 10.1186/1471-2156-4-S1-S100

**Published:** 2003-12-31

**Authors:** Agustin G Yip, Qianli Ma, Marsha Wilcox, Carolien I Panhuysen, John Farrell, Lindsay A Farrer, Diego F Wyszynski

**Affiliations:** 1 Genetics Program, Department of Medicine, Boston University School of Medicine, Boston, Massachusetts, USA

## Abstract

**Background:**

Atherogenic dyslipidemia (AD) is a common feature in persons with premature coronary heart disease. While several linkage studies have been carried out to dissect the genetic etiology of lipid levels, few have investigated the AD lipid triad comprising elevated serum triglyceride, small low density lipoprotein (LDL) particles, and reduced high density lipoprotein (HDL) cholesterol levels. Here we report the results of a whole-genome screen for AD using the Framingham Heart Study population.

**Results:**

Our analyses provide some evidence for linkage to AD on chromosomes 1q31, 3q29, 10q26, 14p12, 14q13, 16q24, 18p11, and 19q13.

**Conclusion:**

AD susceptibility is modulated by multiple genes in different chromosomes. Our study confirms results from other populations and suggests new areas of potential importance.

## Background

Atherogenic dyslipidemia (AD) is characterized by three lipid abnormalities: elevated serum triglyceride, small low density lipoprotein (LDL) particles, and reduced high density lipoprotein (HDL) cholesterol levels [1]. This lipid triad occurs commonly in persons with premature coronary heart disease [2]. Phenotypically, individuals with AD tend to be obese, insulin resistant, and physically inactive. Many investigators believe that AD is a part and precursor of the metabolic syndrome, which also features proinflammatory and prothrombotic states. Several linkage studies have been carried out to dissect the genetic etiology of lipid levels, both quantitatively and discretely. However, there are few studies on AD, and the definitions and study designs are inconsistent. In the present study, we carried out a whole-genome screen with the aim of identifying susceptibility genes for AD using the Framingham Heart Study population.

## Methods

### Population

In this study, we used the Framingham Heart Study population included in Problem 1 distributed to Genetic Analysis Workshop 13 participants. AD was operationally defined as follows: 1) serum tryglyceride level at or above the 90^th ^percentile for age and sex; and 2) serum HDL cholesterol at or below the 25^th ^percentile for age and sex – as given in NHANES III. Individuals with both high triglycerides and low HDL as defined above were considered "affected", while those with neither were considered "unaffected". Affection status was "unknown" in the rest (i.e., those with high triglycerides only or low HDL only, or whose lipid profiles were missing). LDL values were not included in the data set.

Triglycerides and HDL data are available for only one year (1971) in the original cohort (*n *= 394), and then only for 12 individuals. One individual was found to be affected with the AD, 9 were unaffected, and 384 had unknown status. Triglycerides and HDL data are available on all five occasions (spanning two decades) in the offspring cohort (*n *= 1308). Individuals were classified as having AD if they fulfilled the operational criteria at any occasion (*n *= 119); conversely only individuals who were unaffected on every occasion when data were available were categorized as unaffected (*n *= 598) – the rest had unknown status (*n *= 591).

### Statistical analysis

Two-point parametric linkage analysis was performed using VITESSE 2.0 [3]. Assuming the disease locus being at a given map position, we calculated the likelihood of the data, using a range of different dominant and recessive transmission models, all yielding the same disease prevalence and parameterized as a single variable, the heterozygote penetrance. The strategy of obtaining LOD scores using alternative models of inheritance has been tested successfully in several complex disorders [4,5]. To determine a set of likely regions for AD, nonparametric linkage analysis (NPL) based on the Kong and Cox LOD score derived from the S_all _statistic of GENEHUNTER-Plus version 2.0 [6] was used. Finally, two nonparametric affected sib-pair analyses were performed. Maximum-likelihood estimates of the proportions of sib pairs sharing 0, 1, or 2 alleles identically by descent (IBD) at marker loci were estimated with the routine SIB-MLS of the software GAS 2.0 [7]. This nonparametric statistic is used to test for deviations of these proportions from levels expected under the null hypothesis of no linkage. We also performed Haseman-Elston regression [8] for all marker loci versus the trait using full and half-sib relative pairs as implemented in the software SIB-PAIR [9]. While this sib-pair linkage method was originally developed for dealing with continuous traits, it is also applicable to binary traits [10].

## Results

There were 69 families with one affected member, 14 with two, 5 with three, and 2 with four. In all, 11 markers showed some evidence for linkage in parametric analysis (see Table 1), but no marker showed two-point LOD scores ≥ 2.0 or *p *< 0.001. In the multi-point NPL analysis, there was one Kong & Cox LOD score [6] above 2.0 on 14p (see Table 2). The six chromosomal areas with the highest NPL scores were 3q29, 10q26, 14p12, 14q13, 18p11, and 19q13, but none reached significance (LOD score > 3.5). Under the MLS affected sib-pair analyses, seven markers on six chromosomes had two-point MLS LOD scores = 1.20, which correspond to *p *< 0.01 (see Table 3). The Haseman-Elston regression sib-pair analyses revealed two suggestive markers with *p *< 0.05: GGAA23C07 (1q31) and AFM031XA5 (16q24) (see Table 4).

## Discussion

Our choice of an extreme phenotype based on lipid values had a big impact on the number of families informative for linkage analysis. From an original set of 90 families with at least one affected individual, 21 were informative. Due to this limited sample size, we carried out statistical analyses using four alternative approaches that take advantage of the different family structures that were available: affected/unaffected parametric LOD score analysis, affected relative pair NPL analysis, affected sib-pair MLS analysis, and the sib-pair Haseman-Elston regression analysis. Some evidence for linkage to AD was found on chromosomes 1q31, 3q29, 10q26, 14p12, 14q13, 16q24, 18p11, and 19q13. However, due to the limited statistical power, these results should be interpreted with caution.

Several chromosomal areas showed some evidence of linkage by more than one statistical method: 1p (parametric and MLS), 1q (parametric and Haseman-Elston), 14q (NPL and MLS), 16q (parametric and Haseman-Elston), and 19q (parametric and NPL). The latter, 19q13, is particularly important given that this is the area of the ApoC2/ApoE/ApoC1/ApoC4 gene cluster. Several studies have reported significant LOD scores for components of the metabolic syndrome in this region. For example, Elbein and Hasstedt [11] reported a LOD score of 3.16 for triglycerides and of 2.76 for the triglycerides-to-HDL cholesterol ratio in individuals with diabetes. Additionally, the findings on 3q29 and 10q26 are in the vicinity of the suggestive regions reported by Francke et al. [12] in Indo-Mauritians with the metabolic syndrome (3q27, LOD score = 2.13, *p *< 0.001; 10q23, LOD score = 2.06, *p *= 0.001) and of Vionnet et al. [13] in French whites with type 2 diabetes (the markers on 10q26 were also reported by Wiltshire et al. [14] in a UK population with type 2 diabetes). The 16q24 area overlaps with those reported by Soro et al. [15] in Finnish families with low HDL and Aouizerat et al. [16] in Dutch families with familial combined hyperlipidemia.

## Conclusion

Atherogenic dyslipidemia susceptibility is modulated by multiple genes on different chromosomes. Our analyses provide some evidence for linkage to atherogenic dyslipidemia on chromosomes 1q31, 3q29, 10q26, 14p12, 14q13, 16q24, 18p11, and 19q13. Our study confirms results from other populations and suggests new areas of potential importance.

## Figures and Tables

**Table 1:** Two-point parametric LOD scores.

**Chromosome**	**Marker (Location in cM)**	**Lod Score (θ)**	**Model (Penetrance)**
1	GGAT2A07 (45.33)	1.29 (0.10)	Recessive (0.50)
1	GATA65H06 (72.59)	1.26 (0.00)	Dominant (0.75)
1	GATA4H09 (218.46)	1.29 (0.15)	Recessive (0.25)
1	GGAA23C07 (239.66)	1.58 (0.10)	Dominant (0.85)
6	AFM242zg5 (146.06)	1.20 (0.10)	Recessive (0.75)
6	GATA184A08 (166.39)	1.42 (0.10)	Recessive (0.25)
8	GATA72C10 (37.04)	1.29 (0.00)	Dominant (0.85)
10	GATA73E11 (59.03)	1.26 (0.10)	Recessive (0.25)
12	GATA63D12 (95.03)	1.15 (0.00)	Dominant (0.85)
16	AFM031XA5 (113.52)	1.95 (0.10)	Recessive (0.85)
19	Mfd238 (100.61)	1.34 (0.05)	Recessive (0.25)

**Table 2:** Multipoint NPL scores, their *p*-values, and Kong and Cox LOD scores.

**Chromosome**	**Location**	**NPL (*p*-value)**	**K&C LOD**^A^
3	246.82	1.95 (0.028)	1.10
10	179.05	2.36 (0.011)	1.95
14	0.0	2.10 (0.020)	2.01
14	37.92	1.87 (0.033)	0.66
18	31.42	1.71 (0.047)	0.90
19	111.74	1.712 (0.046)	0.74

^A^See Kong and Cox [6].

**Table 3:** Two-point maximum LOD score (MLS).

**Chromosome**	**Marker (Location in cM)**	**MLS**
1	GGAT2A07 (45.33)	1.20
2	GATA69E12 (90.82)	1.20
3	GATA6G12 (209.41)	1.20
13	ATA26D07 (82.93)	1.20
14	ATA29G03 (40.68)	1.20
17	AFM044xg3 (116.86)	1.20
17	AFM217yd10 (126.46)	1.20

**Table 4:** Haseman-Elston regression sib-pair analysis.

**Chromosome**	**Marker (Location in cM)**	**Full Sibs**	**Half Sibs**	**t-Value**	**Asymptotic *p*-Value**
1	GGAA23C07 (218.46)	418	12	3.0	0.0013
16	AFM031XA5 (113.52)	402	12	2.5	0.0064
